# The Real Triumph of Japan or the Conquest of the Silent Foe1Address delivered at the annual meeting of the Association of Military Surgeons, at Detroit, Mich., September 25-29, 1905.

**Published:** 1905-11

**Authors:** Louis L. Seaman

**Affiliations:** New York, 247 Fifth Avenue


### The Real Triumph of Japan or the Conquest of the Silent Foe.[1]

By MAJOR LOUIS L. SEAMAN, M. D., New York.

THE success of Japan in the recent conflict with Russia is due pre-eminently to three fundamental causes: first, thorough preparation and organisation for war—such preparation as was never made before; second, to the simple, non-irritating, and easily digested ration of the Japanese troops; and, third, to the brilliant part played by the members of the medical profession in the application of *practical sanitation,* the stamping out of preventable diseases in the army, thereby saving its units for the legitimate purposes of war—the smashing of the enemy in the field.

It must never be forgotten that in every great campaign an army faces two enemies; first the armed forces of the opposing foe with its various machines for human destruction, that is met at intervals, in open battle; and second, the hidden foe, always found lurking in every camp—the grim specter, ever present, that gathers its victims while the soldier slumbers in hospital, in barrack, or in bivouac—the far greater and silent foe, disease.

Of these enemies the history of warfare for centuries has

1. Address delivered at the annual meeting of the Association of Military Surgeons, at Detroit, Mich., September 25-29, 1905.

proven that in most prolonged campaigns the first, or open enemy, kills twenty per cent. of the total mortality in the conflict, whilst the second or silent enemy kills eighty per cent. In other words, out of every one hundred men who fall in war, twenty die from bullets or wounds, while eighty perish from disease, most of which is preventable. This dreadful and unnecessary sacrifice of life, especially in conflicts between the Anglo-Saxon races, is the most ghastly proposition of the age, and the Japanese have gone a long way toward conquering or eliminating it.

Without minimising for a moment the splendor of her victories on land or sea—Mukden, Port Arthur, Liaoyang, or with Togo in the Korean Straits— and two of these battles are the bloodiest in the annals of history, I yet unhesitatingly assert that the greatest conquests of Japan have been in the humanities of war—in the stopping of this needless sacrifice of life by preventable disease; and gentlemen, the medical men of the army did it.

Longmore's tables, which are accepted as the most reliable statistics of war and which are based on the records of battles for the past 200 years, show that there has rarely been a conflict in which at least four men have not perished from disease for one from bullets. In the Turko-Russian war, in 1878, 80,000 men died from disease and 20,000 from wounds. It is asserted on eminent French authority that in six months of the Crimean campaign, the allied forces lost 50,000 from disease and 2,000 from bullets. A gentleman who remembers that campaign, an ex-president of the New York Academy of Medicine, told me that he had seen whole regiments die away from disease, without ever being on the firing line. In our war with Mexico, the proportion of losses was about four from disease to one from bullets, and in our great Civil War about the same figures were maintained. In round numbers, of the 500,000 fatalities on both sides in that conflict about three-quarters resulted from disease.

There are men living, some of them perhaps present, who may remember that nearly as many men perished from fevers and intestinal diseases in the trenches before Chickahominy as were afterwards slaughtered in the terrible battles that ended our great fratracidal conflict.

No lessons seem to have been learned from these frightful experiences, for later statistics show no improvement. In the French campaign in Madagascar in 1894, 14,000 men were sent to the front, of whom 29 were killed in action and 7,000 perished from preventable disease. In the Boer war in South Africa, the English losses from disease were simply frightful, greater than even our Civil War record. But the crowning piece of imbecility was reserved for our late war with Spain, where more than ten, or to quote a recognised military authority Captain Trout, seven and one-half—(both are horrible enough, and either will suit my purpose for the comparison), were needlessly sacrificed to ignorance and incompetency for every one who died on the firing line or from bullets. This, too, in the short campaign, the actual hostilities of which lasted only six weeks.

The Japanese themselves, in their war with China in 1894, lost about the same average as we did in our rebellion, nearly four from disease for one from bullets, and 45 per cent. of their army suffered from kakki, or beri-beri, rendering them non-effective for the firing line.

All of these statistics were studied with the minutest care and detail by the Japanese. Their authorities recognised that, in order to be victorious over a foe like Russia, this great silent enemy that slaughters 80 out of every 100 that fall, must be overcome. And the medical men of the army *did it.*

The actual figures of killed and wounded and sick in the Japanese army from February, 1904, to the end of April, 1905, are as follows:

| | NUMBER. | PER CENT. |
|---|---|---|
| Killed on field | 43,892 | 7.32 |
| Wounded | 145,527 | 24.27 |
| Died of wounds | 9,054 | 1.51 |
| Sick, including other wounds, accidents, etc., *not* on firing line | 162,556 | 27.11 |
| Died of sickness and disease | 7,435 | 1.24 |
| Contagious cases | 10,563 | 1.93 |
| Died of contagious diseases | 4,557 | .76 |
| Total of dead, wounded and sick | 383,584 | 64.14 |

Note these startling totals:

| | |
|---|---|
| Killed and died from wounds | 52,946 |
| Died from all diseases | 11,992 |

or more than four deaths from bullets for one from disease as against the record of centuries of four from disease to one from bullets; or 800 per cent. better than the average in history.

This represents the entire army in the field, and percentages represent the number of men out of each 100 dead, sick or wounded.

There were 36 men out of every 100 who went to war who were never wounded or sick a day during a year and half's campaign. Only one and two-tenths per cent. of the entire army died of sickness or disease. Only one and one-half per cent. died of gunshot wounds, though 24 per cent. were wounded.

There were 2,000 more men died of wounds than from preventable diseases. Of a total mortality from all causes, of 64,938, there were 40,934 more from casualties than from disease.

This record I believe is unparalleled, and *the medical men of the army achieved it.*

How was this marvelous result attained? Ten years ago, when Japan was robbed of the legitimate fruits of her victory over China by the concerted action of Russia, Germany and France, on the ground of their maintaining the integrity of Chinese territory, and immediately afterwards saw these grasping vultures deliberately appropriating the territory themselves, she recognised the magnitude of her own danger, and set about to prepare for the inevitable struggle that was to determine whether she was to remain an independent nation, or was to become a vassal of the aggresive Muscovite. Her statesmen reasoned in this way: They said, we are about to engage in a terrible war with an antagonist of great strength and prestige, with enormous resources and a supposedly invincible army. That is our first or open enemy in the field. We are also to engage with another enemy, the grim specter that kills eighty out of every hundred who fall in war—this is our second, or hidden foe. Our mortality in the conflict may reach a million men, and it is a sacrifice we are willing to make to preserve our freedom and our institutions. If this terrible slaughter occurs, and the average of the wars of the last two hundred years are maintained, two hundred thousand men will fall on the firing line or from wounds and 800,000 will die in hospitals from disease. For every man who dies there will be at least ten who will be ill, some of whom will be permanently invalided and incapacitated as fighting units. These men will require nursing and hospital care, necessitating enormous expense and impedimenta. We are willing to sacrifice the million men, but the element of the disease with its terrible cost and impedimenta must be *eliminated.*

With this point always in view, she sent her students all over the world to study the army systems in other lands. With the knowledge thus garnered, she evolved a system of her own, based on the practices in vogue in Germany, but greatly modified, and the motto of which might have been "Prevention, not Treatment." She organised her medical department on broad generous lines and gave its representatives the rank and power their greatest responsibilities merit, recognising that they had to deal with a foe that kills eighty per cent. of the total mortality. She even had the temerity (strange as it may seem to an English or American army official) to grade her medical men as high as the officers of the line, who combat the enemy that kills only

20 per cent., and to accord them equal authority—except, of course, in the emergency of battle, when all authority devolves, as it should, on the officers of the line. In her home land she organised the most splendid system of hospitals that have ever been devised for the treatment of sick and wounded, and with her army at the front she put into execution the most elaborate and effective system of sanitation that has ever been practised in war. Upon the declaration of war she was prepared to house, scientifically treat and tenderly care for 25,000 sick and wounded in Japan alone. Twelve sets of main hospitals, each with from one to five attached branch hospitals, were scattered throughout the empire in the chief towns of the 12 military districts into which the country is divided. In other words, the peace footing organisation of the hospital service provided for one main hospital and necessary branches at the headquarters of each army divison.

The original 25,000 odd beds were rapidly increased in number as the campaign progressed, by the erection of substantial, though exceedingly plain pine buildings running parallel and so constructed that each was a unit, housing 100 patients but connected in series by covered walks and runways.

Great elasticity was gained by this simple form of architecture, for wards could be tacked on indefinitely within the limitations of the property area. Each ward was practically isolated, yet for administrative purposes the whole was as one building. Surgical, general medical, contagious and infectious ward series were wholly isolated from one another, by erecting three completely separated series on the same plot of land, each series containing its calculated proportion of unit wards for the specific class of cases for which it was designed.

One and a half years after the commencement of the war, or on the 6th of July, 1905, the twelve great military home hospitals possessed a normal capacity of 58,263 available beds. On this same day, however, only one half of them were in use, or, to be exact, there were 28,561 patients in hospital. Unofficially, but by good authority, I have been informed that the increase in the number of hospitals and beds was made from time to time upon figures deduced from other wars, and the provisions made were thought at first to represent what would be a true relationship of the sick and wounded to the entire force in the field.

The apparent hospital over-preparedness suggests that the Japanese themselves failed to realise what marked successes would attend the enforcement of their new code of military hygiene and sanitation, as applied for the first time in the field.

That it was not really over-preparedness was demonstrated after the battle of Mukden, when the total extraordinary hospital capacity of some 80,000 beds, secured by crowding, was taxed almost to its limits by these shattered phalanxes which poured in by thousands from every transport. It is hardly likely that the military authorities could have foreseen that the Japanese-Russian war would develop the greatest recorded battles of the world, with unparalleled movements of fighting soldiers, and a sacrifice of men by wounds so tremendous that even the spectator on the battlefield fortunately fails to grasp the overwhelming horror. Whether the medical department prepared this immense hospital system for sick or wounded is of little importance; the fact, however being, that when the gastly cortége from Mukden did arrive in Japan in April there was hospital room for every disabled man of the thousands and thousands, and instant medical attendance and care and nursing ready and waiting.

Time does not permit of a detailed description of even one branch of one of these admirably managed military hospitals, but to illustrate the careful detail which is exercised in each department, I may say that they are directed by trained painstaking specialists, and that the most advanced ideas in medicine and surgery are practised in them.

To illustrate one feature, attention might be called to scientific massage which has been developed to a degree in Japanese military treatment never before attempted. Massage is a very old institution in Japan, and with the recent advancement made in the precise knowledge of the body, the skilled masseur has been able to develop and adopt a system of real muscle and nerve stimulation of the utmost importance in military surgery.

A complete text book has been written by the military officer in charge, on the subject of massage largely on the basis of new knowledge acquired in treating thousands of cases during the last 18 months in this hospital. About 25 patients are treated at a time by skilled masseuses under the eye of several technically trained experts, who examine the cases and explain to the kneaders the result which it is desired to attain. The work is done in drill form, *i. e.,* the masseuse at word of command begins operations, works for five minutes, rests two, then continues five more. Ten minutes is usually the limit for a single treatment, though the same patient may come on for several treatments each day.

The large class of surgical gymnastics at this hospital is exceedingly interesting in that the drills are mostly in the open and make a spectacular display. Every man with crippled joints, wasted or contracted muscles, or other physical deformities that can be aided by specialised exercise, is enrolled as a member of the class. Calisthenics of various kinds are indulged in by the men under orders, but the class instead of making all the same movements will be making those of particular advantage in each case. Parallel bars, horizontal bars, swinging rings, stair steps in series of varying heights, cranks, horizontal, vertical and twisting handles, obstacle bars, hurdles and several other devices are arranged in a pretty little grove, and here the crippled go through the motions by which it is hoped to bring them back to normal physical standards.

The pharmaceutical side of these military hospitals is an auxiliary machine, working in perfect harmony with the whole. Like the field service it is indisputably responsible for all the medical and surgical supplies, and issues them upon requisition of the doctors and surgeons. Besides this, the department is responsible for all sterilised milk, washing of bandages and re-rolling, disenfection of bedding, and the making of chemical tests of urine.

Every hospital throughout Japan and every base and field hospital in Manchuria has its bacterological laboratory.

Too much cannot be said in enthusiastic commendation of this side of the service. Undoubtedly the painstaking researches made day by day, even hour by hour, by the corps of trained experts with this instrument, that the dread phantom of disease might be intercepted, has been the means of saving thousands of lives by forestalling possible epidemics, and saving individual life by prompt determination of the trouble. No man suffers from temperature, but whose blood goes under the microscope. Malaria is malaria, and typhoid is typhoid in the Japanese army, and not the Algerian fever, caused by inappropriate and irritating rations, because every case there is differentiated under the microscope and otherwise. Diseases are not guessed at, as they were in Cuba, the Philippines and South Africa, where often for a full week the physicians attempted to diagnose cases by sleight of hand and trick of eye. One wishes to dodge the deluge of shame which shocks us at the remembrance that we, a nation proud of our civilisation and advanced scientific methods, killed thousands of our men through defective organisation and brutal, if not criminal incompetency of those in executive position, while our friends the Japanese, just awakening from so-called barbarism, an oriental, almond-eyed race, which we have hitherto patronised, has shown us that with proper forethought, system and skill, men need not, in appalling numbers, rot and die horribly in the trenches from disease.

Not content with fine bacteriological laboratories in every hospital, one often finds several of the doctors and surgeons carrying on private researches in their own special wards, with microscopes and appliances which are their own private property, preparing slides, raising cultures and working ever, to find the new elusive bacillus whose discovery is to bring them special recognition and fame. When one considers the meagerness of the salaries which these Japanese scientists receive, and the further fact that most of them are poor men, it is to realise that the sacrificing perseverance exhibited, and the enthusiastic love of scientific work shown, will take these people a long journey further on the road of unsummed knowledge.

The limits of this paper do not admit of more than the merest reference to the splendid system of sanitation followed in the field—a specially dangerous field, too, because the water supplies in the territory where the campaign was conducted, had been left infected with the deadly germs of typhoid, dysentery and cholera by the retreating Russians; nor of the water tests and universal use of boiled water for drinking purposes; the physical training of the unit from barrack to battlefield; and the care exercised over his baths, his sleep and his rations. Sufficient to say that during the campaign extending over a year and a half, with from 300,000 to 600,000 soldiers undergoing the severest hardships and privations of active service, there are in the Japanese army 36 men out of every 100 who have never reported at sick call! 36 men who never saw the inside of a hospital or were sick in quarters,—a record absolutely unparalleled. In every other recorded campaign it is found that usually once during a period of every three to five months, each soldier in his organisation, or an average of that number, has reported to the military medical officer for treatment.

I have just returned from the headquarters of the Second Imperial army on the Mongolian frontier, commanded by General Oku, where this year, at least, I found the busiest instrument in the campaign was not the Murata rifle, but the monocular microscope. My opportunities for observation were unexcelled, as the Imperial government in its extreme courtesy accorded me all the privileges of a foreign medical attaché; and weeks were spent in the military hospitals of Japan prior, and subsequent to my visit to the front. The war has taught many lessons and destroyed many ideals in matters military, as in matters surgical, where the heretofore accepted idea of the duties of the military surgeon has been shown to be altogether erroneous, where asepis and antisepsis have relegated the use of the scalpel to comparative obscurity, and demonstrated most conclusively that preservation of the army by prevention of disease is the surgeon's duty, first, last and nearly all the time. In surgical technique, or in the after treatment of the wounded and sick, the Japanese have taught the foreigner comparatively little, but in the field of sanitary science and dietetics they have demonstrated, what has never been done before, that preventable diseases *are* preventable, and that the grim specter, which lingers in every barrack, tent and quarter, and which in all great wars of history has been responsible for nearly 80 out of every 100 of the recorded deaths, can be controlled. They have demonstrated that the great incubus of an army in the field, the presence of crowded hospitals and the large and expensive force neccessary to equip and conduct them, can, to a large extent, be eliminated. They have preserved their armies for the legitimate purposes for which armies are enlisted,—the killing or conquering of an open enemy in the field, instead of having four-fifths of its mortality victims to the silent foe.

It is against this dreadful scourge, this needless sacrifice, that the Japanese have made their hardest fight and won their most signal victories—victories which will redound more to their credit, than even the expulsion of the Muscovite aggressor.

In the matter of discipline, Captain Tanake, aide-de-camp of Marshal Oyama, told me at Mukden, there had been but 12 court-martials in the Manchurian army, the majority of which were for cruelties to Chinamen. The number of suicides during the war was 86, the majority being those who were refused permission to accompany the colors to Manchuria, on account of some physical defect, and the remainder, because they preferred death to capture by the enemy. There has been but one desertion—a fanatical pharmacist—who was caught by the screen of the Japanese near Mukden, disguised as a Chinese coolie, and concealing on his person a quantity of poison. He imagined himself the deliverer of Japan, and attempted to gain admission to the presence of Kuropatkin, as a servant, for the purpose of poisoning him, as he regarded Kuropatkin as the bitterest enemy of his country. He was court-martialed and severly punished. Was there ever an army with such a record?

A prominent officer of the United States regular army who had never been through Japan or in Manchuria, said to me recently: "The medical service of the Japanese is inferior to that of the Americans; that no doctor had any business at the front; that if one of them appeared on the firing line or near it, when he was in command, he would kick him back to his place in the hospital where he should remain looking after the sick and the wounded and attending to the business that he was paid for; and if he refused to go he would put him under arrest and have him court-martialed; that all these examinations of water and wells and streams were humbug and tomfoolery, and that the use of boiled water on marches or in camp was impractical. In times of war, he said, the place for a doctor was behind the army, taking care of the sick and wounded, but never in the front. If a doctor objected to a situation selected by him for an encampment on the ground of its unsanitary condition or bad water supply, he would tell the doctor to go to hell." This man, too, had been detailed as an instructor in one of our large universities. I would not quote his utterances were it not for the fact that he reflects a sentiment of a class of officers whose knowledge of sanitation scarcely rivals that of a mud turtle; and that he illustrates the type of a man most dangerous to the safety of the army and to the nation.

A despatch received in London on September 21 from the Tokio correspondent of the London *Standard*, giving some of the statistics of the war to that date, reports: "Killed 46,180, died of wounds 10,970, died from sickness 15,300. This percentage of death to sickness was less than one-fourth of the total dead, which is a record not paralleled in the annals of war."

When contemplating these marvelous figures, with what a ghastly and melancholy smile the hero of Manila must recall his action in censoring the cablegram of the Chief Surgeon who had requested 50 additional medical officers and 200 more nurses when the hospital wards were overcrowded, because such a dispatch would prove the falsity of his claim that he had "the situation well in hand." Months afterwards the surgeons and nurses were provided, but not until the horrible condition was intensified, and taps had sounded the requiem for many a poor boy who had joined the great majority.

Perhaps the same delight may solace the contemplative commander in the Cuban campaign, when he recalls his famous order at Tampa, directing the unloading of a ship filled with medical and hospital supplies for Santiago, and the substitution of a load of mules instead.

Or of another major-general during that war, who, on being waited upon by certain medical officers with a protest against the use of certain water said, in response to their complaint: "When I want your advice, I will send for you; until I do, you can attend to your own business."

Or even if the then Secretary of War, who, when inspecting the camps at Montauk Point with the President of the United States, said on looking at a glass of water furnished the troops of this infected camp, and which certain medical men had pronounced to contain germs of disease: "Why, it looks all right to me."

Gentlemen, is our great profession a profession that in one of the bloodiest wars of history has demonstrated its ability to reduce the mortality of deaths from disease 800 per cent.,—is it to remain subservient to the dictates of this variety of judgement; or is the medical department of the army to be reorganised on rational lines and empowered to enforce its mandates?

How sanitary matters were regulated by the authorities in Japan, may be seen from the following: On the tenth of May last, Count Katsura, Premier of the Empire, said to me, "we are in constant fear of the approaching summer, lest these most dreaded enemies, cholera, dysentery and typhoid fever, left by the retreating foe, excite epidemics in our ranks, and it is against them that our most strenuous efforts are now being directed. If our army could campaign in a new country, undefiled by former occupants, its danger would be greatly reduced." * * * *

Until the line and staff officer of the American army is taught the necessity of sanitation, and the medical officer is given rank and authority to enforce it, our medical department must remain a humiliating failure. Its continuance under present conditions is no less than an evidence of national imbecility.

The wife of an American officer, whose life had been needlessly sacrificed through preventable disease in one of our island possessions, returning from the sad scene, recently said to me: "Those who love their country are willing to give their dearest and best for their country's good, but there is little glory and much suffering for the soldier who dies from disease in a foreign clime, wasting his life instead of losing it in the defense of his country's honor. We, the mothers, must teach our children the lessons of loyalty; must imbue the boy of today with the patriotism that will make him the soldier of tomorrow. We may dwell with loving pride on the glory of death on the firing-line, and inculcate in him the lessons of heroism which characterised his father's life and made his death a triumph. But what can we say to the son of the man who has died from disease due to the lack of care by the country for which he was willing to sacrifice his family's interests and happiness, and his own life? Loyalty has its birth in love, and its death in hate. This is not the first nor the last war America will have to fight, and it behooves our lawmakers to look well to the care of the soldier of today so as to count on the sons for tomorrow, for America's future depends on the lessons her sons are now learning."

247 Fifth Avenue.

